# Comparative Anti-inflammatory Activity of a Nanocomposite-Based Herbal Oral Rinse and a Commercial Oral Rinse

**DOI:** 10.7759/cureus.61548

**Published:** 2024-06-02

**Authors:** Anjusha Divakar, Remmiya Mary Varghese, Aravind Kumar S, Rajeshkumar Shanmugam

**Affiliations:** 1 Orthodontics and Dentofacial Orthopedics, Saveetha Dental College and Hospitals, Saveetha Institute of Medical and Technical Sciences, Saveetha University, Chennai, IND; 2 Nanobiomedicine Lab, Centre for Global Health Research, Saveetha Medical College and Hospital, Saveetha Institute of Medical and Technical Sciences, Saveetha University, Chennai, IND

**Keywords:** nanocomposite-mediated oral rinse, herbal oral rinse, biocompatible, commercial oral rinse, anti-inflammatory agent

## Abstract

Introduction: The present study aimed to evaluate and compare the anti-inflammatory effects of two oral rinse formulations, a commercial oral rinse and an *Ocimum tenuiflorum *and *Ocimum gratissimum* (nanocomposites, NCs) oral rinse, using in vitro assays commonly employed to assess anti-inflammatory activity.

Materials and methods: The anti-inflammatory potential of the oral rinse formulations was assessed using bovine serum albumin (BSA) denaturation, egg albumin denaturation, and membrane stabilization assays. Diclofenac sodium was used as a reference standard in all assays. The inhibition percentages of BSA denaturation and egg albumin denaturation assays, as well as membrane stabilization effects, were measured at various concentrations of the oral rinse formulations.

Results: Both the commercial oral rinse and the *Ocimum tenuiflorum* and *Ocimum gratissimum* oral rinse demonstrated significant inhibition of BSA denaturation, indicating their anti-inflammatory potential. The *Ocimum tenuiflorum* and *Ocimum gratissimum* (NCs) oral rinse consistently showed higher inhibition percentages than the commercial oral rinse, suggesting stronger anti-inflammatory effects in this assay. In the egg albumin denaturation assay, both formulations exhibited inhibition of protein denaturation, with the *Ocimum tenuiflorum* and *Ocimum gratissimum* (NCs) oral rinse showing comparable or slightly higher inhibition percentages. The membrane stabilization assay further supported the anti-inflammatory properties of both formulations, with the *Ocimum tenuiflorum* and *Ocimum gratissimum* (NCs) oral rinse demonstrating efficacy comparable to diclofenac sodium.

Discussion: The results suggest that *Ocimum tenuiflorum* and *Ocimum gratissimum* (NCs) oral rinse may possess stronger anti-inflammatory effects compared to commercial oral rinse, as evidenced by higher inhibition percentages in the BSA denaturation assay. Both formulations showed promising anti-inflammatory activity in the egg albumin denaturation and membrane stabilization assays, indicating their potential for mitigating inflammation.

Conclusion: The *Ocimum tenuiflorum* and *Ocimum gratissimum* (NCs) oral rinse exhibits significant anti-inflammatory effects in vitro, potentially surpassing the efficacy of the commercial oral rinse. Further studies are needed to explore the clinical implications of these findings and to validate the anti-inflammatory properties of the *Ocimum tenuiflorum* and *Ocimum gratissimum* (NCs) oral rinse in vivo.

## Introduction

The use of nonsteroidal anti-inflammatory drugs (NSAIDs) in dentistry for managing pain and swelling can be associated with various side effects [1]. These side effects can range from mild issues such as nausea to more severe problems such as gastric bleeding or perforation [2]. NSAIDs can also increase the risk of vascular accidents, renal toxicity, and abnormal bleeding due to their antiplatelet effect. However, studies suggest that the risk of acute myocardial infarction associated with NSAIDs is higher in long-term users compared to occasional users, making their use in dental practice generally safe [3]. Nanotechnology allows for the development of nanomaterials, such as nanospheres, nanotubes, and nanocomposites (NCs), that enable precise and targeted delivery of anti-inflammatory drugs within the oral cavity. This approach minimizes the systemic side effects commonly associated with conventional drug administration [4].

Nanoparticle-based formulations enhance the bioavailability and effectiveness of anti-inflammatory agents, leading to better management of inflammation and pain using lower drug doses [5]. Localized delivery of anti-inflammatory drugs using nanotechnology reduces the risk of systemic side effects, which are often seen with traditional NSAIDs. Nanotechnology-based methods, such as using nanocarriers and scaffolds, support periodontal tissue regeneration [6]. This approach targets the root causes of inflammation, offering potential long-term benefits beyond symptom management. Certain nanoparticles, such as silver and zinc oxide, possess inherent antimicrobial properties, aiding in controlling oral bacterial load and potentially reducing reliance on anti-inflammatory medications [7,8].

Nano-based oral rinses present an intriguing approach to addressing inflammation in the oral cavity. By incorporating nanoparticles, these oral rinses can precisely target areas such as the gingival sulcus, delivering anti-inflammatory agents directly to sites of inflammation. Nano-based oral rinses can focus on specific areas of inflammation, such as the gingival sulcus, optimizing the delivery of anti-inflammatory agents where they are needed most [9]. For instance, a seawater-based oral rinse has been studied for its effectiveness in reducing plaque and gingivitis compared to chlorhexidine. Natural compounds are also being explored as alternatives to chlorhexidine, showing promise in managing dental plaque and gingivitis [10]. These oral rinses can incorporate antimicrobial nanoparticles, such as silver-based compounds, to control bacterial growth in the oral cavity. This dual action can help combat inflammation and reduce the bacterial load simultaneously [11,12].

Nano-based oral rinses hold potential benefits over systemic anti-inflammatory drug administration, offering targeted and controlled delivery that may enhance treatment outcomes while minimizing systemic side effects [13]. As research in this area progresses, these innovative products could play a valuable role in managing inflammation associated with periodontal disease and other oral health conditions [14]. In this present study, an oral rinse was prepared using an NC of silver and zinc oxide, in which *Ocimum grattissimum* and *Ocimum tenuiflorum* extracts were used as reducing and capping agents. A commercial oral rinse was also used to evaluate their anti-inflammatory activity using bovine serum albumin denaturation, egg albumin denaturation, and membrane stabilization assays.

## Materials and methods

Preparation of herbal formulation

A solution was formulated by precisely combining 1 g of both *Ocimum tenuiflorum* and *Ocimum gratissimum* extracts with 100 mL of distilled water. The mixture was subjected to heating at 60 °C for 15-20 minutes using a heating mantle. Subsequent to the boiling process, the mixture underwent gradual filtration using filter paper. The resultant filtrate, which harbored the extract, was subsequently stored for the synthesis of nanoparticles.

Green synthesis of ZnONPs and AgNPs

The green synthesis of zinc oxide nanoparticles (ZnONPs) and silver nanoparticles (AgNPs) was conducted in this research utilizing African basil and Black tulsi extracts (*Ocimum tenuiflorum* and *Ocimum grattissimum*) in the presence of a zinc nitrate solution (30 mM in 50 mL distilled water) and a 1 mM silver nitrate solution, respectively. The bioactive compounds present in the herbal extracts were harnessed to reduce and stabilize the nanoparticles. Initially, a controlled source of zinc ions was provided by preparing a zinc nitrate solution. Subsequently, a mixture of 50 mL of African basil and Black tulsi extract, known for their rich phytochemical content, was mixed with the zinc nitrate solution.

For the synthesis of AgNPs, a 1 mM silver nitrate solution was prepared by dissolving silver nitrate in 80 mL of distilled water, followed by the addition of 20 mL of a filtered herbal formulation extract. The resulting mixtures were subjected to centrifugation at 8000 revolutions per minute (rpm) for 10 minutes. The centrifugation step played a pivotal role in both the ZnONPs and AgNPs synthesis processes by facilitating the separation of the synthesized nanoparticles from any unreacted precursors or extract residues. The collected pellet after centrifugation contained the desired ZnONPs and AgNPs, which were subsequently characterized and evaluated.

Green synthesis of silver and zinc oxide NCs (Ag+ZnONCs)

The synthesis of silver and zinc oxide NCs (Ag+ZnONCs) through a green approach involved the combination of equal volumes of 2 mL from the obtained pellets of silver (Ag) and zinc oxide (ZnO) nanoparticles. This amalgamation was carried out using a magnetic stirrer set at a rotation speed of 600 rpm. The objective of this procedure was to ensure comprehensive dispersion and homogenization of the two types of nanoparticles, thereby facilitating their interaction and integration into the structure of the NC. The stirring operation was sustained for a period of five to six hours to allow ample time for the nanoparticles to amalgamate into a unified NC. Subsequently, the synthesized Ag+ZnONC pellet was collected and transferred for subsequent processing.

Preparation of Ag+ZnONCs-based oral rinse

The preparation of an oral rinse based on Ag+ZnONCs involved combining 0.3 g of sucrose, 0.1 g of sodium lauryl sulfate, 0.001 g of sodium benzoate, and 500 µL of Ag+ZnONCs in 10 mL of distilled water. Sucrose was utilized as a sweetening agent, sodium lauryl sulfate as a foaming agent, and sodium benzoate as a preservative. The resulting mixture underwent thorough mixing to produce a green-synthesized NC-based oral rinse.

Anti-inflammatory activity

The green-synthesized silver and zinc oxide NC-based oral rinse and commercial oral rinse were comparatively tested for their anti-inflammatory activity using three assays, including BSA denaturation, egg albumin denaturation, and membrane stabilization assays.

BSA denaturation assay

A solution containing 0.45 mL of BSA was prepared by mixing it with 0.05 mL of green-synthesized silver and zinc oxide NC-based mouthrinse and commercial mouthrinse, which were present in various concentrations ranging from 10 to 50 µg/mL. Subsequently, the pH of the solution was adjusted to 6.3. The mixture was then incubated at room temperature for a duration of 10 minutes. Following this, it was subjected to a 30-minute incubation period in a water bath at 55 °C. For comparison purposes, diclofenac sodium was utilized as the standard group, while dimethyl sulfoxide served as the control. Finally, the samples were analyzed spectrophotometrically at a wavelength of 660 nm.

The percentage of protein denaturation was determined utilizing the following equation: % inhibition = (absorbance of control - absorbance of sample × 100)/absorbance of control.

Egg albumin denaturation assay

For the egg albumin denaturation assay, a reaction mixture was prepared by mixing 0.2 mL of fresh egg albumin with 2.8 mL of phosphate buffer. To this mixture, green-synthesized silver and zinc oxide NC-based mouthrinse and commercial mouthrinse were added in varying concentrations, ranging from 10 to 50 µg/mL. The pH of the solution was then adjusted to 6.3. The mixture was subsequently incubated at room temperature for a period of 10 minutes. Following this, it was subjected to a 30-minute incubation period in a water bath at 55 °C. For comparison, diclofenac sodium was employed as the standard group, whereas dimethyl sulfoxide was utilized as the control. Finally, the samples were analyzed spectrophotometrically at a wavelength of 660 nm.

The percentage of protein denaturation was determined utilizing the following equation: % inhibition = (absorbance of control - absorbance of sample × 100)/absorbance of control.

Membrane stabilization assay

The in vitro membrane stabilization assay evaluated the membrane-stabilizing properties of the compounds. This assay assessed the potential of the green-synthesized silver and zinc oxide NC-based oral rinse and commercial oral rinse (10-50 µg/mL) to prevent the disruption of cell membranes and the subsequent release of intracellular contents. The assay utilized tris-HCl buffer, human red blood cells (RBCs), phosphate-buffered saline (PBS), centrifuge tubes, and a UV-visible spectrophotometer.

Preparation of RBC suspension

Fresh human blood was collected in a sterile tube with anticoagulants. After centrifuging the blood at 1000 g for 10 minutes at room temperature, the RBCs were separated. The RBCs were washed three times with PBS and resuspended in tris-HCl buffer to create a 10% (v/v) RBC suspension.

Assay procedure

Each centrifuge tube was filled with 1 mL of the RBC suspension, followed by the addition of different concentrations of NC-based oral rinse and commercial oral rinse. The tubes were gently mixed and incubated at 37 °C for 30 minutes. After centrifuging the tubes at 1000 rpm for 10 minutes at room temperature, the absorbance of the supernatant was measured at 540 nm using a UV-visible spectrophotometer. The percentage inhibition of hemolysis was calculated as follows: % inhibition = (OD control - OD sample)/OD control × 100.

Here, OD control is the absorbance of the RBC suspension without the test compound(s), and OD sample is the absorbance of the RBC suspension with the test compound.

## Results

BSA denaturation assay

**Figure 1:**
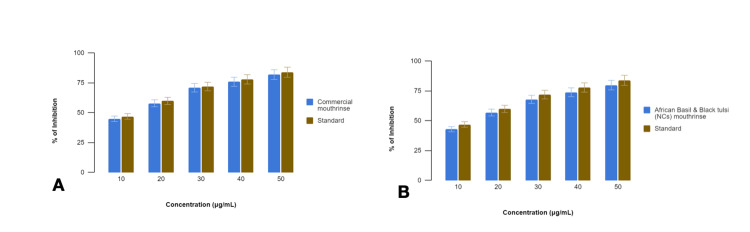
Comparison of anti-inflammatory activity of (A) commercial oral rinse and (B) African basil and Black tulsi (silver and zinc oxide nanocomposite) oral rinse using bovine serum albumin denaturation assay

In this study, we investigated the anti-inflammatory properties of two oral rinse formulations, a commercial oral rinse and an African basil and Black tulsi (NCs) oral rinse, using a BSA denaturation assay. Diclofenac sodium was employed as a standard reference for comparison (Figure 1).

The commercial oral rinse demonstrated significant inhibition of BSA denaturation, indicative of anti-inflammatory activity. The percentage inhibition of BSA denaturation by the commercial mouthrinse at various concentrations (10%, 20%, 30%, 40%, and 50% v/v) was 45%, 58%, 71%, 76%, and 82%, respectively. In comparison, diclofenac sodium exhibited inhibition percentages of 47%, 60%, 72%, 78%, and 84% at the corresponding concentrations. The commercial oral rinse consistently displayed slightly lower inhibition of BSA denaturation compared to diclofenac sodium across all tested concentrations.

Similarly, the African basil and Black tulsi (NCs) oral rinse showed significant anti-inflammatory activity as evidenced by the inhibition of BSA denaturation. The percentage inhibition by African basil and Black tulsi (NCs) mouthrinse at the tested concentrations was 43%, 57%, 68%, 74%, and 80%. Diclofenac sodium, used as a standard, displayed higher inhibition percentages of 47%,60%, 72%, 78%, and 84% at the corresponding concentrations. The African basil and Black tulsi (NCs) oral rinse consistently exhibited lower inhibition compared to diclofenac sodium across all concentrations.

The results of the BSA denaturation assay indicate that the African basil and Black tulsi (NCs) oral rinse exhibits higher anti-inflammatory effects than the commercial oral rinse, with inhibition of BSA denaturation observed at varying levels across concentrations. However, both oral rinse formulations showed slightly lower efficacy compared to diclofenac sodium under the experimental conditions. These findings underscore the potential anti-inflammatory properties of the tested oral rinse formulations and suggest avenues for further investigation into optimizing their efficacy and mechanisms of action.

Egg albumin denaturation assay

**Figure 2:**
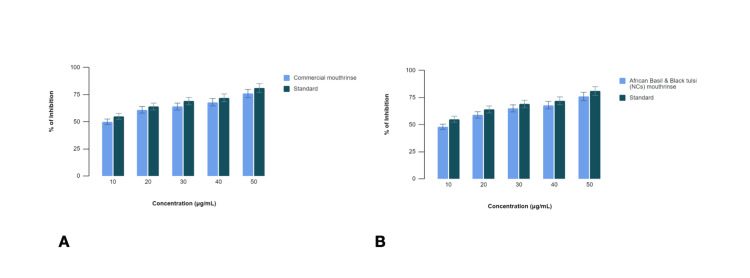
Comparison of anti-inflammatory activity of (A) commercial oral rinse and (B) African basil and Black tulsi (silver and zinc oxide nanocomposite) oral rinse using egg serum albumin denaturation assay

In this study, we assessed the anti-inflammatory potential of two oral rinse formulations, a commercial oral rinse and an African basil and Black tulsi (NCs) oral rinse, using an egg albumin denaturation assay. Diclofenac sodium served as the standard reference for comparison (Figure 2).

The commercial oral rinse exhibited varying levels of inhibition of egg albumin denaturation compared to diclofenac sodium across different concentrations (10%, 20%, 30%, 40%, and 50% v/v). The percentage inhibition by the commercial mouthrinse at each concentration was as follows: 10%-50%, 20%-61%, 30%-64%, 40%-68%, and 50%-76%. In comparison, diclofenac sodium demonstrated inhibition percentages of 55%, 64%, 69%, 72%, and 81% at the corresponding concentrations. The commercial oral rinse displayed slightly lower inhibition compared to diclofenac sodium across all concentrations tested.

Similarly, the African basil and Black tulsi (NCs) oral rinse showed inhibition of egg albumin denaturation compared to diclofenac sodium. The percentage inhibition by the African basil and Black tulsi (NCs) oral rinse at the tested concentrations was 10%-48%, 20%-59%, 30%-65%, 40%-68%, and 50%-76%. Diclofenac sodium, used as a standard, exhibited inhibition percentages of 55%, 64%, 69%, 72%, and 81% at the corresponding concentrations. The African basil and Black tulsi (NCs) oral rinse displayed comparable inhibition to diclofenac sodium across most concentrations tested.

Both the commercial oral rinse and African basil and Black tulsi (NCs) oral rinse demonstrated inhibition of egg albumin denaturation, indicating potential anti-inflammatory effects. While the inhibition levels varied slightly between the oral rinse formulations and diclofenac sodium, the African basil and Black tulsi (NCs) oral rinse showed comparable efficacy to the standard at several concentrations.

Membrane stabilization assay

**Figure 3:**
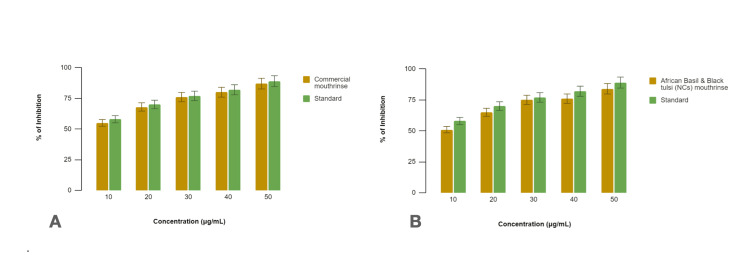
Comparison of anti-inflammatory activity of (A) commercial oral rinse and (B) African basil and Black tulsi (silver and zinc oxide nanocomposite) oral rinse using membrane stabilization assay

In this study, we evaluated the membrane stabilization effects of two oral rinse formulations, a commercial oral rinse and an African basil and Black tulsi (NCs) oral rinse, using a membrane stabilization assay. Diclofenac sodium was used as the standard reference for comparison (Figure 3).

The commercial oral rinse demonstrated membrane stabilization effects compared to diclofenac sodium across various concentrations (10%, 20%, 30%, 40%, and 50% µg/mL). The percentage membrane stabilization by the commercial oral rinse at each concentration was as follows: 10%-51%, 20%-65%, 30%-75%, 40%-76%, and 50%-84%. In comparison, diclofenac sodium exhibited membrane stabilization percentages of 58%, 70%, 77%, 82%, and 89% at the corresponding concentrations. The commercial oral rinse showed slightly lower membrane stabilization efficacy compared to diclofenac sodium across most concentrations tested.

Similarly, the African basil and Black tulsi (NCs) oral rinse demonstrated membrane stabilization compared to diclofenac sodium. The percentage membrane stabilization by the African basil and Black tulsi (NCs) oral rinse at the tested concentrations was 10%-55%, 20%-68%, 30%-76%, 40%-80%, and 50%-87%. Diclofenac sodium, used as a standard, exhibited membrane stabilization percentages of 58%, 70%, 77%, 82%, and 89% at the corresponding concentrations. The African basil and Black tulsi (NCs) mouthrinse displayed comparable membrane stabilization efficacy to the standard across several concentrations.

Both the commercial oral rinse and African basil and Black tulsi (NCs) oral rinse demonstrated membrane stabilization effects, indicative of their potential anti-inflammatory properties. While the membrane stabilization efficacy varied slightly between the oral rinse formulations and diclofenac sodium, the African basil and Black tulsi (NCs) mouthrinse showed comparable effects to the standard at multiple concentrations. These findings suggest that both NC-based oral rinse formulations possess membrane-stabilizing properties, supporting their potential therapeutic applications for oral inflammation.

## Discussion

The present study aimed to evaluate and compare the anti-inflammatory effects of two oral rinse formulations, a commercial oral rinse and an African basil and Black tulsi (NCs) herbal oral rinse, using several in vitro assays commonly employed to assess anti-inflammatory activity. In the BSA denaturation assay, both the commercial oral rinse and the African basil and Black tulsi (NCs) herbal oral rinse demonstrated significant inhibition of BSA denaturation, indicative of their anti-inflammatory potential. While both formulations exhibited lower inhibition percentages compared to diclofenac sodium across all concentrations, the African basil and Black tulsi (NCs) oral rinse consistently showed higher inhibition percentages than the commercial oral rinse. This suggests that the African basil and Black tulsi (NCs) oral rinse may possess stronger anti-inflammatory effects in this assay.

Similarly, in the egg albumin denaturation assay, both mouthrinse formulations displayed inhibition of egg albumin denaturation, signifying their ability to mitigate protein denaturation associated with inflammation. The African basil and Black tulsi (NCs) oral rinse again exhibited comparable or slightly higher inhibition percentages compared to the commercial mouthrinse across most concentrations tested [14]. The membrane stabilization assay further supported the anti-inflammatory properties of both the commercial oral rinse and the African basil and Black tulsi (NCs) oral rinse. Both formulations demonstrated membrane stabilization effects, with the African basil and Black tulsi (NCs) oral rinse showing a comparable efficacy to diclofenac sodium across several concentrations [15].

Commercial oral rinse products are widely used for oral hygiene purposes, but like any medication or oral care product, they may be associated with certain side effects. Mouth irritation, including a burning sensation, discomfort, or sensitivity in the oral tissues (such as gums, tongue, or inner cheeks), is a common side effect reported with some commercial mouthrinses. This irritation can be caused by ingredients such as alcohol, antimicrobial agents, or flavoring agents present in the mouthrinse formulations. Studies have noted that certain oral rinse formulations containing alcohol or other strong antimicrobial agents may contribute to oral mucosal irritation and discomfort [15].

In some cases, the use of commercial oral rinse may lead to swollen glands (lymph nodes) on the side of the face or neck. Swollen glands can be a rare side effect and may indicate an immune response or localized inflammation triggered by components within the oral rinse formulation. Research has suggested that certain ingredients in oral rinse products, such as chlorhexidine, may contribute to lymphadenopathy or swelling of lymph nodes [16]. Another less common side effect associated with commercial oral rinse use is irritation of the tongue tip. This can present as redness, soreness, or discomfort localized to the tip of the tongue. Tongue tip irritation may be related to direct contact with oral rinse ingredients or alterations in oral microbiota following oral rinse use. Studies have also highlighted the potential for oral rinse ingredients to cause adverse reactions in sensitive individuals, including tongue irritation [17].

The observed stronger anti-inflammatory effects of the NC formulation of African basil and Black tulsi mouthrinse compared to commercial mouthrinse can be attributed to several mechanisms that enhance the bioavailability and efficacy of its bioactive phytochemicals. The formulation of African basil and Black tulsi extracts into an NC structure likely improves the solubility and dispersibility of bioactive compounds, such as phenolic compounds and flavonoids. Nanoscale particles enhance the surface area available for interaction with solvents, leading to better dissolution and dispersion of these compounds, ultimately making them more readily available for absorption and exerting their anti-inflammatory effects [18].

The nanosized structure of the NC formulation facilitates better permeability of bioactive compounds across biological membranes. This enhanced permeability allows for improved absorption and distribution of the phytochemicals in the oral cavity, increasing their bioavailability and overall effectiveness in combating inflammation. The NC matrix can provide a controlled and sustained release of bioactive compounds. This controlled release profile ensures prolonged exposure of the compounds at the site of action (i.e., oral tissues), maintaining therapeutic concentrations over an extended period. Consequently, this sustained release contributes to the sustained anti-inflammatory effects observed with African basil and Black tulsi (NCs) mouthrinse [19].

The NC formulation acts as a protective barrier, shielding bioactive compounds from premature degradation. By preventing degradation, the NC matrix maintains the stability and integrity of the phytochemicals, preserving their bioactivity and enhancing their overall bioavailability within the oral cavity [20,21]. The NC formulation enables targeted delivery of bioactive compounds to specific sites of inflammation in the oral cavity. The small particle size and surface properties of NCs can facilitate the active targeting of inflamed tissues, maximizing therapeutic efficacy while minimizing systemic exposure and potential side effects [22,23].

Implications and potential mechanisms

The observed anti-inflammatory effects of the African basil and Black tulsi (NCs) mouthrinse are particularly noteworthy. African basil (*Ocimum gratissimum*) and Black tulsi (*Ocimum tenuiflorum*) are known for their pharmacological properties, including anti-inflammatory and antioxidant activities attributed to their rich phytochemical composition, such as phenolic compounds and flavonoids. The NC formulation may enhance the bioavailability and efficacy of these bioactive compounds, contributing to the observed anti-inflammatory effects. While the commercial oral rinse also demonstrated anti-inflammatory activity in the assays, the slightly lower efficacy observed compared to the African basil and Black tulsi (NCs) mouthrinse suggests that the latter may offer enhanced therapeutic potential for oral inflammation management.

Limitations

It is essential to acknowledge the limitations of this study, including the use of in vitro assays that may not fully replicate the complex inflammatory processes occurring in vivo. Future research should focus on conducting in vivo studies to validate the anti-inflammatory effects observed in this study and explore the safety and efficacy of African basil and Black tulsi (NCs) oral rinse in clinical settings. Overall, the findings of this study highlight the potential of African basil and Black tulsi (NCs) oral rinse as a promising anti-inflammatory agent for oral health applications.

## Conclusions

The present study compared the anti-inflammatory effects of two mouthrinse formulations, a commercial oral rinse and an African basil and Black tulsi (NCs) oral rinse, using various in vitro assays. Both formulations exhibited significant inhibition of protein denaturation and membrane stabilization, indicating their potential anti-inflammatory properties. The African basil and Black tulsi (NCs) oral rinse consistently showed higher inhibition percentages in the BSA denaturation assay and comparable or slightly higher inhibition percentages in the egg albumin denaturation assay compared to the commercial oral rinse. These findings suggest that the African basil and Black tulsi (NCs) oral rinse may possess stronger anti-inflammatory effects than the commercial oral rinse. Overall, both formulations demonstrated promising anti-inflammatory activity, with the African basil and Black tulsi (NCs) oral rinse showing potential as a natural alternative with comparable efficacy to diclofenac sodium. Further research is warranted to explore the clinical implications of these findings and to validate the anti-inflammatory effects of the African basil and Black tulsi (NCs) oral rinse in vivo.
